# Advances in the management of osteopenia: a case series

**DOI:** 10.1186/s13256-025-05816-9

**Published:** 2026-02-18

**Authors:** Maurizio Nordio, Roberto Baldelli

**Affiliations:** 1 Department of Experimental Medicine, Sapienza University of Rome, Rome, Italy; 2 The Experts Group on Inositol in Basic and Clinical Research and on PCOS (EGOI-PCOS), Rome, Italy; 3 Endocrinology Unit, Department of Oncology and Medical Specialties, A.O. San Camillo-Forlanini, Rome, Italy

**Keywords:** Case report, Osteopenia, Calcium, Vitamin D, Vitamin K, d-chiro-inositol

## Abstract

**Background:**

Osteopenia is a pre-pathological condition characterized by reduced bone mineral density that may lead to increased fracture risk and to the onset of osteoporosis. Standard treatments for managing osteopenia include supplementation of calcium, vitamin D, and vitamin K. Interestingly, recent studies suggest d-chiro-inositol and α-lactalbumin may enhance bone health through mechanisms such as insulin sensitization and improved nutrient absorption; furthermore, d-chiro-inositol is thought to be a specific mediator of osteoclast activity by inhibiting the expression of several osteoclastogenic genes.

**Case presentation:**

Six Caucasian patients with osteopenia—four men and two women with an average age of about 41 years—were treated with a daily regimen of calcium (400 mg), cholecalciferol (50 μg), vitamin K2 (50 μg), d-chiro-inositol (150 mg), and α-lactalbumin (30 mg) for 2 months. Even though the results reported no significant changes in T-score value, all patients exhibited improved serum levels of vitamin D and osteocalcin, along with fluctuations in alkaline phosphatase and parathyroid hormone levels.

**Conclusion:**

Despite the preliminary nature of the obtained data, combined supplementation exerted positive effects on bone metabolism by influencing serum levels of both vitamin D and osteocalcin, thus suggesting enhanced bone formation. Biochemical improvements support the potential of d-chiro-inositol and α-lactalbumin, in addition to traditional supplements, as a therapeutic strategy for managing osteopenia, even though further clinical studies are needed to clarify the effects and related molecular mechanisms.

## Introduction

Osteopenia is a pre-pathological condition defined as the precursor of osteoporosis and characterized by reduced bone mineral density (BMD). It is a serious threat to bone health owing to the occurrence of increasing susceptibility to fractures and bone-related diseases. In clinical practice the diagnosis of such condition relies on the use of a patient’s T-score, which is a statistical representation of the value of BMD. In particular, a BMD value between −1.0 and −2.5 indicates a condition of osteopenia, while lower values indicate a condition of osteoporosis [1, 2]. Given its detrimental effect on human health, it is important to understand and monitor the risk factors associated with osteopenia, which include tobacco use, alcohol abuse, vitamin D and calcium deficiency, and pathological low values of body mass index (BMI) [3, 4]. Furthermore, several concomitant pathologies may contribute to increase the risk of developing osteopenia, including kidney and liver disease, celiac disease, rickets, type I diabetes mellitus, hyperparathyroidism, hypogonadism, and amenorrhea. In addition, alterations in thyroid functionality may impact bone metabolism: evidence in literature highlighted that hyperthyroidism may contribute to the onset of osteoporosis, while hypothyroidism may impede bone remodeling [5, 6]. Indeed, thyroid hormones play a central role in skeletal development regulating growth, bone mass, and maturation, and are important regulators of bone maintenance in adults [7].

Moreover, the use of several pharmaceuticals, including corticosteroids, levothyroxine, immunosuppressors, and gonadotropin-releasing hormone (GnRH) analogs, may worsen the risk of developing osteopenia [4]. In particular, the use of GnRH analogs is relevant as it suppresses estrogen production, which is a crucial element in bone health that promotes osteoblast differentiation and bone deposition [8].

Bone remodeling is a continuous process physiologically driven by the activity of osteoclasts, which resorb bone mass, and the activity of osteoblasts, which form bone mass, thus ensuring maintenance of bone strength and mineral homeostasis; however, in case of osteopenia, this balance is disrupted with an unbalanced activity of osteoclasts. Key biomarkers in bone health and metabolism include parathormone (PTH), osteocalcin, and alkaline phosphatase (ALP), among others, all of which influence bone turnover rate. PTH is a hormone produced by parathyroid glands that regulates circulating levels of calcium and phosphorus; osteocalcin is a protein produced by osteoblasts whose levels are indicative of bone formation rate; ALP is an enzyme primarily produced by osteoblasts that is involved in bone mineralization. Understanding these underlying processes is crucial for the development of targeted interventions that aim to restore bone density or prevent further progression to osteoporosis [9, 10].

Typically, the clinical management of osteoporosis includes pharmaceuticals such as bisphosphonates, monoclonal antibodies, and PTH analogs; besides this, supplements such as calcium, vitamin D, and vitamin K, have well-documented roles within bone health [2, 11, 12]. In addition, recent evidence has highlighted the potential role of d-chiro-inositol (DCI), along with its analog pinitol (3-o-methyl-d-chiro-inositol), in supporting bone health. Typically used as insulin sensitizers or hormone regulators, evidence in literature reported these compounds as exhibiting promising effects in increasing bone density and positively influencing bone metabolism [13]. Given the effect on insulin, DCI supplementation helps prevent hyperglycemia, which may weaken bone collagen, thus compromising bone strength [14]. Moreover, Liu *et al*. [15] demonstrated the effectiveness of DCI treatment in counteracting bone loss in a study model of ovariectomized mice, which are unable to produce estrogens and therefore are at higher risk of losing BMD. Indeed, the authors demonstrated that d-pinitol promotes an increased BMD in these mice by increasing the DCI content in bones. Of note, in a separate study, Yu *et al*. highlighted that *in vitro* treatments with DCI inhibit the maturation of osteoclasts by influencing the expression of several osteoclastogenic genes through the down modulation of NFATc1, a transcriptional factor belonging to the signaling pathway of RANK/RANKL [14].

On the basis of the aforementioned scientific evidence, herein we report a series of case studies derived from clinical experience, where we supplemented patients with osteopenia with calcium 400mg, cholecalciferol 50μg (corresponding to 2000 IU of vitamin D), vitamin K_2_ (menaquinone) 50μg, DCI 150mg, and α-lactalbumin (α-LA) 30mg. Patients were recommended to take the treatment once per day for 2 months. After this period, we evaluated T-score value and blood levels of ALP, osteocalcin, vitamin D, and PTH, thus comparing each value with the corresponding baseline.

## Case presentation

Between August and November 2023, six Caucasian patients—four men and two women with an average age of about 41 years—presented at our outpatient clinic for a routine check-up of thyroid nodules and underwent a comprehensive endocrine evaluation. Given the impact of thyroid functionality on bone health, patients also underwent quantitative heel analysis—or quantitative ultrasound (QUS) of the heel—which is a method using ultrasound to assess bone quality and predict fracture risk, often used as a screening tool for osteoporosis. Blood levels of thyroid-stimulating hormone (TSH), triiodothyronine (T3), and thyroxine (T4) were in the physiological range, while QUS revealed reduced values of BMD, indicating a clinical condition of osteopenia. Patients were treated following the recommendations of the Italian Ministry of Health, taking calcium and vitamin D supplementation. Recommendations also include reducing alcohol intake and smoking habits. Notably, in combination with the standard medical care, we prescribed the combined supplementation of vitamin D, calcium, vitamin K, DCI, and α-LA for a 2-month period and monitored the variation of their bone health status (Table 1).

**Table 1:** Clinical parameters related to bone health in the evaluated six patients

Patient	T-score		ALP		PTH		Vitamin D		Osteocalcin	
	Baseline	2 month-treatment	Baseline	2 month-treatment	Baseline	2 month-treatment	Baseline	2 month-treatment	Baseline	2 month-treatment
Case 1	−1.7	−1.7	87 U/L	82 U/L	21.3 pg/mL	24.2 pg/mL	39.4 ng/mL	54.8 ng/mL	25.6 ng/mL	27.1 ng/mL
Case 2	−1.6	−1.6	40 U/L	55 U/L	39.2 pg/mL	37.7 pg/mL	21.3 ng/mL	25.3 ng/mL	16.6 ng/mL	17.7 ng/mL
Case 3	−1.4	−1.4	45 U/L	53 U/L	41.0 pg/mL	40.0 pg/mL	27.4 ng/mL	36.5 ng/mL	13.3 ng/mL	21.3 ng/mL
Case 4	−1.3	−1.3	40 U/L	36 U/L	58.8 pg/mL	58.4 pg/mL	38 ng/mL	44.7 ng/mL	15.8 ng/mL	21.3 ng/mL
Case 5	−1.3	−1.3	35 U/L	42 U/L	45.7 pg/mL	48.2 pg/mL	45.4 ng/mL	46.1 ng/mL	37.6 ng/mL	41.4 ng/mL
Case 6	−1.5	−1.5	83 U/L	92 U/L	51.4 pg/mL	52.4 pg/mL	25.6 ng/mL	32.4 ng/mL	20.4 ng/mL	21.9 ng/mL

This table reports values of the evaluated clinical parameters that are related to bone health. The first column of each parameter indicates values at baseline, while the second column indicates values after 2 months of treatment with calcium, vitamin D, vitamin K, DCI, and α-LA

Case 1 was a 37-year-old man, non-smoker, non-drinker. The patient exhibited no change in T-score after 2 months of treatment, remaining stable at −1.7, thus indicating no changes in bone density. Biochemical markers reported that ALP levels decreased marginally from 87 to 82 U/L, while osteocalcin levels increased from 25.6 to 27.1 ng/mL. Vitamin D levels increased from 39.4 to 54.8 ng/mL, and PTH levels reported a slight increase from 21.3 to 24.2 pg/mL.

Case 2 was a 53-year-old perimenopausal woman, smoker (> 10 cigarettes per day), non-drinker. The patient reported a T-score of −1.6 at the beginning of the study that did not change following 2 months of treatment. However, there was a notable increase in serum levels of ALP from 40 to 55 U/L and a slight increase in osteocalcin levels from 16.6 to 17.7 ng/mL, alongside a minor improvement in vitamin D levels from 21.3 to 25.3 ng/mL following treatment. In addition, PTH levels decreased from 39.2 to 37.7 pg/mL.

Case 3 was a 37-year-old man, non-smoker, non-drinker. During the treatment the patient experienced no change in T-score value, which remained stable at −1.4 throughout the oral assumption. However, following treatment, levels of ALP rose from 45 to 53 U/L and those of osteocalcin from 13.3 to 21.3 ng/mL. Vitamin D levels rose from 27.4 to 36.5 ng/mL, and PTH levels remained relatively stable, decreasing slightly from 41.0 to 40.0 pg/mL.

Case 4 was a 42-year-old woman, non-smoker, non-drinker. In this case, the patient maintained a stable T-score value of −1.3, with ALP levels decreasing from 40 to 36 U/L and osteocalcin increasing from 15.8 to 21.3 ng/mL. Vitamin D levels improved from 38 to 44.7 ng/mL, and PTH levels shifted from 58.8 to 58.4 pg/mL.

Case 5 was a 35-year-old man, non-drinker, light smoker. Following treatment, this patient maintained a stable T-score value of −1.3, with a rise in ALP from 35 to 42 U/L. The patient also exhibited an increase in osteocalcin levels from 37.6 to 41.4 ng/mL. Vitamin D levels exhibited a minimal improvement from 45.4 to 46.1 ng/mL, and PTH levels increased from 45.7 to 48.2 pg/mL.

Case 6 was a 44-year-old man, non-smoker, non-drinker. Following treatment, the patient demonstrated no change in T-score value, which remained at −1.5. ALP levels increased from 83 to 92 U/L, while osteocalcin levels exhibited a modest rise from 20.4 to 21.9 ng/mL. Vitamin D levels improved from 25.6 to 32.4 ng/mL, and PTH levels increased from 51.4 to 52.4 pg/mL.

## Discussion and conclusions

The presented clinical cases indicate a positive response to the combined supplementation regimen, based on calcium, vitamin D, vitamin K, DCI, and α-LA, with all patients showing improvements in serum levels of both vitamin D and osteocalcin. We considered healthy values as follows: osteocalcin in men 4.6–65.4 ng/mL, osteocalcin in women 6.5–42.3 ng/mL, and vitamin D3 > 30 ng/mL. Three out of six patients displayed healthy values of vitamin D at baseline (> 30 ng/mL), while the remaining three had values between 20 and 30 ng/mL but exhibited no alterations in the other parameters.

No changes were detected either in T-score or in BMD value at the conclusion of the treatment, likely owing to the brief period of 2-month supplementation. However, osteocalcin levels rose in all the patients, possibly suggesting a beneficial effect when coupled with an increase in vitamin D levels. The data on serum levels of vitamin D are in line with previous studies; while the increase in osteocalcin levels observed after our treatment regimen is in contrast with previous studies [16]. In this context, we posited that the improved serum levels of osteocalcin could likely be attributed to the use of DCI and vitamin K that appears to have a positive impact on bone deposition. Of course, the limited number of patients included in these clinical cases is a crucial element of discussion.

We observed fluctuations in the levels of ALP and PTH, both of which are essential players in bone health and metabolism. Notably, all patients showed healthy values prior to and following treatment for both ALP (33–98 U/L) and PTH (15–68.3 pg/mL). The observed fluctuations appear to be incidental, rather than a result of the treatment. This suggests that patients had no impairments derived from altered parathyroid activity or phosphate metabolism in bones. Nonetheless, prior to treatment, four of the six cases displayed ALP values near the lower threshold, while three patients had PTH levels in the upper half of the interval. In contrast, following treatment, five out of six cases displayed ALP levels closer to the optimal values.

Previous literature supports the use of DCI in bone metabolism, suggesting its supplementation in combination with standard medical care in the case of osteopenia [14, 15]. Furthermore, a prior study observed that clinical treatment with vitamin K reduces the amount of undercarboxylated osteocalcin, which is inversely related to BMD [17]. Considering the above-mentioned evidence, these molecules could represent a beneficial boost to the current available treatments for osteopenia. Therefore, the potential synergic therapeutic effect of DCI with calcium, vitamin D, and vitamin K warrants further clinical investigation with a larger population and longer treatments, also including a follow-up period of analysis.

Considering that this is a case report series with a diverse group of patients, further support to our findings may be derived from pooled data analysis, even though we are aware that the clinical cases are different for sex, age, and habits. When regrouping and analyzing the data via a non-parametric Wilcoxon signed rank test, we observed a significant increase in vitamin D levels (*p* < 0.05) after 2-month treatment. Most importantly, we found that osteocalcin levels significantly rose (*p* < 0.05), pointing out an improvement in the bone formation of patients following the 2-month treatment. Finally, the changes in both ALP and PTH levels appeared to be nonsignificant. Of course, we are aware of the weakness of the provided statistical analysis considering the limited number of cases and the variability among age and habits, along with the period of treatment. Therefore, further studies with a larger group of subjects are needed to confirm and support these observations. Recent clinical evidence have demonstrated that several patients may exhibit impairment in intestinal absorption of inositols. In recent years, researchers demonstrated that α-LA may regulate the opening kinetics of mammalian intestinal tight junctions, promoting the absorption of micronutrients, including inositols [18–20]. Moreover, α-LA seems to stabilize vitamin D, increasing its bioavailability [21, 22]. In particular, as reported in literature, in its molten globule state at low values of pH, α-LA can bind vitamin D and protect it from degradation, thus improving its solubility and transport.

This case series, while limited by its scope, provides preliminary evidence supporting the combined use of vitamin D, vitamin K, calcium, DCI, and α-LA in the treatment of osteopenia, thus reinforcing the importance of nutritional interventions to preserve bone health. Further clinical research is necessary to confirm our observations and elucidate the mechanisms through which these nutrients exert their positive effects on bone metabolism. Weaknesses of this study include the limited number of patients and the duration of the study protocol, as larger randomized trials are required to fully establish the effect of the combined supplement regimen.

In conclusion, this case study represents the first clinical evidence supporting the potential role of DCI in the field of bone health. The data presented herein, while preliminary, suggests DCI may be effective, in combination with regularly recommended supplements such as calcium and vitamin D, at preserving BMD in patients with osteopenia. While further long-term studies are required to fully evaluate the effect of DCI on BMD and clarify the involved molecular mechanisms, this work suggests that this non-pharmaceutical approach may help slow the progression towards osteoporosis in patients with osteopenia.

## Data Availability

All data generated or analyzed during this study are included in this published article.

## References

[1] Maggi S, Noale M, Giannini S, Adami S, Defeo D, Isaia G, Sinigaglia L, Filipponi P, Crepaldi G. Quantitative heel ultrasound in a population-based study in Italy and its relationship with fracture history: the ESOPO study. Osteoporos Int. 2006;17:237–44. 16142503 10.1007/s00198-005-1985-2

[2] Svedbom A, Hernlund E, Ivergård M, Compston J, Cooper C, Stenmark J, McCloskey EV, Jönsson B, Kanis JA. Osteoporosis in the European Union: a compendium of country-specific reports. Arch Osteoporos. 2013;8:137. 24113838 10.1007/s11657-013-0137-0 PMC3880492

[3] Clynes MA, Harvey NC, Curtis EM, Fuggle NR, Dennison EM, Cooper C. The epidemiology of osteoporosis. Br Med Bull. 2020;133:105–17. 32282039 10.1093/bmb/ldaa005 PMC7115830

[4] Sözen T, Özışık L, Başaran N. An overview and management of osteoporosis. Eur J Rheumatol. 2017;4:46–56. 28293453 10.5152/eurjrheum.2016.048 PMC5335887

[5] Vandenbroucke A, Luyten FP, Flamaing J, Gielen E. Pharmacological treatment of osteoporosis in the oldest old. Clin Interv Aging. 2017;12:1065–77. 28740372 10.2147/CIA.S131023 PMC5505539

[6] Zhu S, Pang Y, Xu J, Chen X, Zhang C, Wu B, Gao J. Endocrine regulation on bone by thyroid. Front Endocrinol. 2022;13: 873820. 10.3389/fendo.2022.873820 PMC9020229 35464058

[7] Williams GR, Bassett JHD. Thyroid diseases and bone health. J Endocrinol Invest. 2018;41:99–109. 28853052 10.1007/s40618-017-0753-4 PMC5754375

[8] Almeida M, Laurent MR, Dubois V, Claessens F, O’Brien CA, Bouillon R, Vanderschueren D, Manolagas SC. Estrogens and androgens in skeletal physiology and pathophysiology. Physiol Rev. 2017;97:135–87. 27807202 10.1152/physrev.00033.2015 PMC5539371

[9] Reid IR, Bristow SM. Calcium and bone. Handb Exp Pharmacol. 2020;262:259–80. 31792679 10.1007/164_2019_324

[10] Zhang Z, Nam HK, Crouch S, Hatch NE. Tissue nonspecific alkaline phosphatase function in bone and muscle progenitor cells: control of mitochondrial respiration and ATP production. Int J Mol Sci. 2021. 10.3390/ijms22031140. 33498907 10.3390/ijms22031140 PMC7865776

[11] Priemel M, von Domarus C, Klatte TO, Kessler S, Schlie J, Meier S, Proksch N, Pastor F, Netter C, Streichert T, *et al*. Bone mineralization defects and vitamin D deficiency: histomorphometric analysis of iliac crest bone biopsies and circulating 25-hydroxyvitamin D in 675 patients. J Bone Miner Res. 2010;25:305–12. 19594303 10.1359/jbmr.090728

[12] Sato T, Inaba N, Yamashita T. MK-7 and its effects on bone quality and strength. Nutrients. 2020. 10.3390/nu12040965. 32244313 10.3390/nu12040965 PMC7230802

[13] Cipriani F, Gnessi L, Watanabe M, Baldelli R. Inositols and bone health: potential therapeutic applications in osteoporosis prevention and treatment. Nutrients. 2025. 10.3390/nu17121999. 40573111 10.3390/nu17121999 PMC12195919

[14] Yu J, Choi S, Park ES, Shin B, Yu J, Lee SH, Takami M, Kang JS, Meong H, Rho J. D-chiro-inositol negatively regulates the formation of multinucleated osteoclasts by down-regulating NFATc1. J Clin Immunol. 2012;32:1360–71. 22711011 10.1007/s10875-012-9722-z

[15] Liu X, He C, Koyama T. d-pinitol ameliorated osteoporosis via elevating d-chiro-inositol level in ovariectomized mice. J Nutr Sci Vitaminol. 2023;69:220–8. 37394427 10.3177/jnsv.69.220

[16] Sohouli MH, Wang S, Almuqayyid F, Gabiatti MP, Mozaffari F, Mohamadian Z, Koushki N, Alras KA, AlHossan AM, Albatati SK, *et al*. Impact of vitamin D supplementation on markers of bone turnover: systematic review and meta-analysis of randomised controlled trials. Eur J Clin Invest. 2023;53: e14038. 37314058 10.1111/eci.14038

[17] Je SH, Joo NS, Choi BH, Kim KM, Kim BT, Park SB, Cho DY, Kim KN, Lee DJ. Vitamin K supplement along with vitamin D and calcium reduced serum concentration of undercarboxylated osteocalcin while increasing bone mineral density in Korean postmenopausal women over sixty-years-old. J Korean Med Sci. 2011;26:1093–8. 21860562 10.3346/jkms.2011.26.8.1093 PMC3154347

[18] Monastra G, Sambuy Y, Ferruzza S, Ferrari D, Ranaldi G. Alpha-lactalbumin effect on myo-inositol intestinal absorption: *in vivo* and *in vitro*. Curr Drug Deliv. 2018;15:1305–11. 29745333 10.2174/1567201815666180509102641

[19] Ranaldi G, Ferruzza S, Natella F, Unfer V, Sambuy Y, Monastra G. Enhancement of D-chiro-inositol transport across intestinal cells by alpha-lactalbumin peptides. Eur Rev Med Pharmacol Sci. 2020;24:10143–54. 33090422 10.26355/eurrev_202010_23234

[20] Kamenov Z, Gateva A, Dinicola S, Unfer V. Comparing the efficacy of myo-inositol plus α-lactalbumin vs. myo-inositol alone on reproductive and metabolic disturbances of polycystic ovary syndrome. Metabolites. 2023. 10.3390/metabo13060717. 37367875 10.3390/metabo13060717 PMC10304700

[21] Delavari B, Saboury AA, Atri MS, Ghasemi A, Bigdeli B, Khammari A, Maghami P, Moosavi-Movahedi AA, Haertlé T, Goliaei B. Alpha-lactalbumin: a new carrier for vitamin D3 food enrichment. Food Hydrocolloids. 2015;45:124–31.

[22] Pedersen JN, Sørensen HV, Otzen DE. Stabilizing vitamin D(3) using the molten globule state of α-lactalbumin. J Dairy Sci. 2018;101:1817–26. 29331461 10.3168/jds.2017-13818

